# Sound-Based Unsupervised Fault Diagnosis of Industrial Equipment Considering Environmental Noise

**DOI:** 10.3390/s24227319

**Published:** 2024-11-16

**Authors:** Jeong-Geun Lee, Kwang Sik Kim, Jang Hyun Lee

**Affiliations:** 1 Department of Smart Digital Engineering, Inha University, Incheon 22212, Republic of Korea; jeonggeun2.lee@doosan.com; 2 Doosan Bobcat Korea Co., Ltd., Incheon 22503, Republic of Korea; 3 Extreme Technology Research Center for Ship and Offshore Platform, Inha University, Incheon 22212, Republic of Korea; kskim@inha.ac.kr; 4 Department of Naval Architecture and Ocean Engineering, Inha University, Incheon 22212, Republic of Korea

**Keywords:** MIMII, forklift, PHM, fault diagnosis, MFCC, STFT, GANs, VAE, DANN, domain adaptation

## Abstract

The influence of environmental noise is generally excluded during research on machine fault diagnosis using acoustic signals. This study proposes a fault diagnosis method using a variational autoencoder (VAE) and domain adaptation neural network (DANN), both of which are based on unsupervised learning, to address this problem. The proposed method minimizes the impact of environmental noise and maintains the fault diagnosis performance in altered environments. The fault diagnosis algorithm was implemented using acoustic signals containing noise, present in the malfunctioning industrial machine investigation and inspection open dataset, and the fault prediction performance in noisy environments was examined based on forklift acoustic data using the VAE and DANN. The VAE primarily learns from normal state acoustic data and determines the occurrence of faults based on reconstruction error. To achieve this, statistical features of Mel frequency cepstral coefficients were extracted, generating features applicable regardless of signal length. Additionally, features were enhanced by applying noise reduction techniques via magnitude spectral subtraction and feature optimization, reflecting the characteristics of rotating equipment. Furthermore, data were augmented using generative adversarial networks to prevent overfitting. Given that the forklift acoustic data possess time-series characteristics, the exponentially weighted moving average was determined to quantitatively track time-series changes and identify early signs of faults. The VAE defined the reconstruction error as the fault index, diagnosing the fault states and demonstrating excellent performance using time-series data. However, the fault diagnosis performance of the VAE tended to decrease in noisy environments. Moreover, applying DANN for fault diagnosis significantly improved diagnostic performance in noisy environments by overcoming environmental differences between the source and target domains. In particular, by adapting the model learned in the source domain to the target domain and considering the domain differences based on signal-to-noise ratio, high diagnostic accuracy was maintained regardless of the noise levels. The DANN evaluated interdomain similarity using cosine similarity, enabling the accurate classification of fault states in the target domain. Ultimately, the combination of the VAE and DANN techniques enabled effective fault diagnosis even in noisy environments.

## 1. Introduction

### 1.1. Background of Study

In industrial machinery, data from various signals, such as vibration, temperature, pressure, and acoustic signals, can be used for feature extraction, and machine learning-based classification algorithms can be applied to detect anomalies and failures in equipment containing rotating and nonrotating components. In particular, deep learning methods such as convolutional neural networks (CNNs) have been widely used to classify such anomalies and failures. However, the problem of domain shift, owing to differences in data distribution between the actual operational and laboratory environments, has attracted significant attention. Fault diagnosis models trained on test data obtained from laboratory experiments may exhibit reduced accuracy in relation to fault classification when applied to equipment operating in actual environments. In particular, the additional noise present in actual environments can considerably degrade the performances of fault diagnosis models. Therefore, the domain shift problem must be addressed by adapting fault diagnosis or anomaly detection algorithms trained in laboratory settings to closely align with actual operating conditions, considering noise and environmental variations. Domain shift can decrease the performance of such models, reducing the accuracy of fault diagnosis. Solving this problem enables reliable fault diagnosis across various environments and ensures the accuracy of fault diagnosis under actual operational settings. The malfunctioning industrial machine investigation and inspection (MIMII) dataset, containing failure indication signals from various environments including rotating and nonrotating equipment, is publicly available and designed to evaluate the domain shift problem [1]. In particular, the front end of a forklift, a critical piece of industrial equipment for loading and unloading cargo, can cause substantial human and material damage when malfunctions occur. Forklift failures are relatively infrequent in actual operational environments; therefore, fault diagnosis models must be prepared using degradation tests and artificially induced failure experiments conducted in laboratory settings. Nevertheless, models trained in laboratory settings may exhibit low accuracy due to noise and environmental differences in actual operational environments. The noise present in actual operational environments is a major source of interference during fault diagnosis. This study proposes a fault diagnosis method that considers the noise and low failure frequency in real operational environments based on forklift fault datasets obtained from laboratory experiments. More specifically, this study addresses two conditions: the absence of failure labels in data acquired from actual operational environments and the presence of background noise. Therefore, unsupervised learning and domain adaptation are key technical considerations in this study.

### 1.2. Literature Review

Prognostics and health management (PHM) uses sensors to monitor the conditions of systems and diagnose and predict abnormal states [2,3]. Features are extracted from the measured sensor signals, and faults are diagnosed or predicted by applying machine learning and deep learning algorithms [4,5,6]. These techniques can be combined with traditional reliability-based equipment-life prediction to estimate the remaining useful life (RUL) of a system based on metrics such as mean time to failure [7,8,9]. However, such statistical methods are limited when accounting for real-time changes in operating environments and equipment conditions; therefore, the use of real-time condition-based PHM techniques is appropriate in many cases [10,11]. Traditional machine learning methods such as support vector machines, k-nearest neighbor, and the Gaussian mixture model (GMM) have been used for fault diagnosis [12,13]. For diagnosing faults in nonrotating machinery, indicators such as acoustic signals, vibrations, or thermal imaging can be used. In particular, acoustic signals provide valuable information for diagnosing faults in nonrotating machinery with low-frequency characteristics. The characteristics of acoustic signals can be analyzed in the time, frequency, or time–frequency domain, enabling the identification of fault types and locations. Signal analysis techniques include Fourier transform, wavelet transform, and the use of Mel frequency cepstral coefficients (MFCCs). MFCCs efficiently extract spectral information from acoustic signals and are widely used in sound recognition and fault diagnosis of machinery [14,15]. Machine learning algorithms, combined with the mentioned signal processing techniques, are used to classify and detect fault features. Deep learning models such as autoencoders, variational autoencoders (VAEs), and CNNs have shown excellent performances in terms of learning data features and detecting fault types through unsupervised learning. CNNs, in particular, are well suited for analyzing unstructured data such as acoustic signals and can effectively learn the frequency patterns of these signals using multi-dimensional filters [16,17].

However, three major challenges related to fault diagnosis remain as follows: (1) environmental noise and domain adaptation issues, (2) gradual degradation and condition changes of equipment, and (3) the absence of fault labels [18,19]. The domain shift problem, in particular, is one of the most serious challenges arising from the differences between data collected in laboratory settings and those collected from actual operational environments. To overcome this challenge, techniques for noise reduction and domain adaptation are being studied, and the MIMII dataset is widely used to evaluate domain shift issues across various environments [20,21,22]. Nguyen et al. [23] converted acoustic signals obtained from the MIMII dataset into Mel spectrograms and diagnosed faults using a CNN-based autoencoder. Furthermore, Müller et al. [24] extracted features using Mel spectrograms and an ImageNet model and compared the fault diagnosis performances of several machine learning algorithms. These methods involve extracting features from Mel spectrogram images or applying log transformation to derive MFCCs [25]. As acoustic data have high-resolution characteristics, a substantial amount of computational time is required to extract features. When diagnosing faults in machinery that is gradually aging, analyzing the progressive changes in the time series of signals is critical [25]. By combining principal component analysis (PCA), Mel spectrograms, and CNNs, the gradual condition changes of equipment can be tracked while considering the temporal variability in acoustic signals. In the absence of normal and fault labels in equipment signals, unsupervised learning techniques such as GMM, k-means clustering, and isolation forest are used for anomaly detection [26,27,28]. Deep learning algorithms such as the VAE and generative adversarial networks (GANs) can be applied to detect anomalies in datasets containing only labels indicating normal states [29]. Specifically, the autoencoder has a flexible strength in feature extraction through unsupervised learning, particularly in noisy, complex signals or constrained environments [30]. These algorithms learn from data containing only normal conditions and subsequently detect abnormalities by measuring the deviation between new inputs and the learned normal state [19,31,32].

Domain adaptation techniques, such as domain-adversarial neural networks (DANNs), are designed to minimize the gap between source and target domains. Additionally, unsupervised domain adaptation techniques exhibit improved fault prediction performances in environments in which labeled data are scarce. For instance, da Costa et al. [33] applied the DANN technique for fault diagnosis and prediction of RUL. Liu and Gryllias [34] proposed a method for enhancing fault prediction performance in relation to rotating bearings via unsupervised domain adaptation. The authors used domain adaptation techniques such as maximum mean discrepancy and correlation alignment to minimize the discrepancy between the source and target domains, thereby addressing the issue of label scarcity and offering a reliable prediction model across various environments. Ganin et al. [35] proposed the domain-adversarial neural network (DANN) method for learning domain-invariant features using image datasets without labeled data. In their architecture, the feature extractor learns domain-invariant features, and a domain classifier is added to predict whether the input data belong to the source or target domain. Through experiments across various domains, they demonstrated that DANN outperforms traditional domain adaptation methods, especially when there is a large discrepancy between the source and target domains. Tzeng et al. [36] advanced DANN by applying adversarial learning techniques to more effectively adjust the features between the source and target domains. Research on fault diagnosis using DANN includes studies by Chen et al. [37], Sicilia et al. [38], Chen et al. [39], and Gentner and Susto [40]. Similarly, the studies conducted by Liu et al. [41] and Wang et al. [42] incorporated domain adaptation techniques for diagnosing faults in rotating equipment. In the study conducted by Wang et al. [42], adversarial learning techniques, such as the gradient adversarial layer (GRL), were employed to effectively reduce the data distribution gap between the source and target domains. Additionally, the use of CNN-based deep learning models enabled the precise classification of faults via the analysis of vibration and acoustic data while maintaining high performance even in actual operational environments containing noise.

The techniques proposed in these studies attempt to diagnose faults in nonrotating forklift equipment, which is also the focus of this study. In particular, the previous studies suggest approaches that consider various environmental conditions and the lack of fault labels in acoustic signal-based fault diagnosis.

### 1.3. Research Objective

This study proposes a method for applying fault diagnosis models, trained in laboratory settings, to actual operational environments. Specifically, forklift equipment is considered, for which obtaining fault labels and histories is challenging, and a reliable fault diagnosis model that can perform well even in noisy environments is proposed. The applicability of unsupervised learning techniques, including the VAE, and domain adaptation methods, such as the DANN, is first explored. This represents a key approach for addressing the domain shift problem identified in the literature to overcome the discrepancies between laboratory data and data obtained from actual operational environments, and an efficient fault diagnosis method that reflects various physical characteristics is proposed. Using the MIMII dataset and experimental data from nonrotating equipment, this study analyzes fault data under different operational environments and combines deep learning techniques such as CNNs, VAEs, and GANs to address issues related to data imbalance and noise. Based on this, a fault diagnosis model that accounts for noise and environmental variability in real-time settings is developed. Additionally, this study explores the potential of GAN-based data augmentation techniques to address the scarcity of fault data, and noise reduction techniques are applied to filter noise. Furthermore, the study proposes appropriate methods for capturing the gradual changes in fault states by reflecting the progressive changes in time-series characteristics.

The key issues addressed in this study are as follows:

1. Data preprocessing and feature extraction considering noise.
2. Methods for efficiently extracting pattern changes from time-series data.
3. Application of diagnostic models considering the domain shift problem.
4. The use of unsupervised learning models for anomaly detection by learning only normal state data.
5. The application of fault diagnosis methods reflecting the physical characteristics of both nonrotating and rotating equipment.

Finally, this study proposes a methodology to apply fault diagnosis models trained in laboratory settings to real operational environments, overcoming the domain shift problem and maintaining fault diagnosis accuracy even in noisy environments.

### 1.4. Structure of the Paper

**Figure 1:**
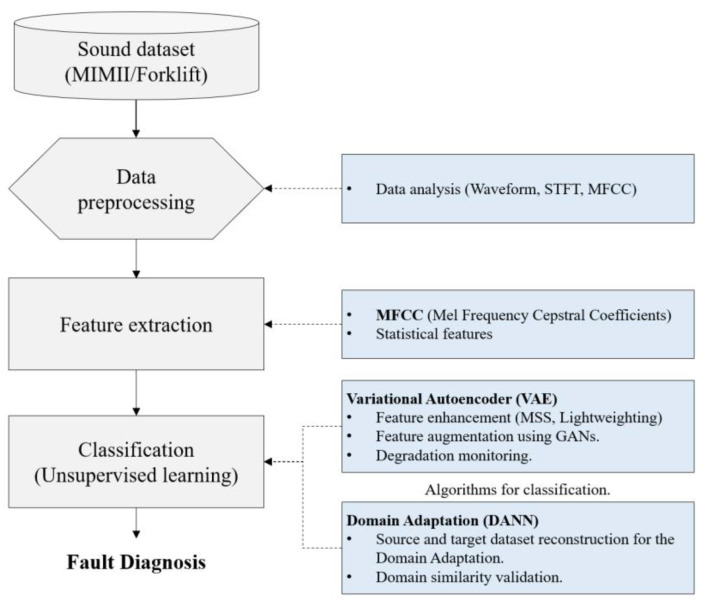
Feature extraction and fault diagnosis procedure using the MIMII and forklift datasets.

The fault diagnosis procedure proposed in this study is briefly presented in Figure 1, and the contents of this paper are organized as follows:

- Section 2 introduces the MIMII and forklift datasets and the preprocessing procedure used on both datasets. The signal processing and statistical feature extraction processes are presented. Furthermore, the application of extracted features to fault diagnosis algorithms is explained, and the effectiveness of these algorithms is verified via one-dimensional (1D)-CNN analysis.
- Section 3 presents a fault diagnosis method based on a fully connected VAE model. Considering the time-series characteristics of the forklift dataset, reconstruction error is used as a fault index, and the exponentially weighted moving average (EWMA) is determined to detect equipment degradation and hidden failures.
- Section 4 discusses the application of domain adaptation techniques for solving the domain shift problem between the MIMII and forklift datasets in detail. The similarity analysis and feature extraction between the source and target domains are explained, and a 1D-CNN-based feature extractor is introduced to enhance computational efficiency.
- Section 5 presents the experimental results, the performance analysis of the proposed methods, and the conclusions of the study.

## 2. Dataset and Feature Extraction

**Figure 2:**
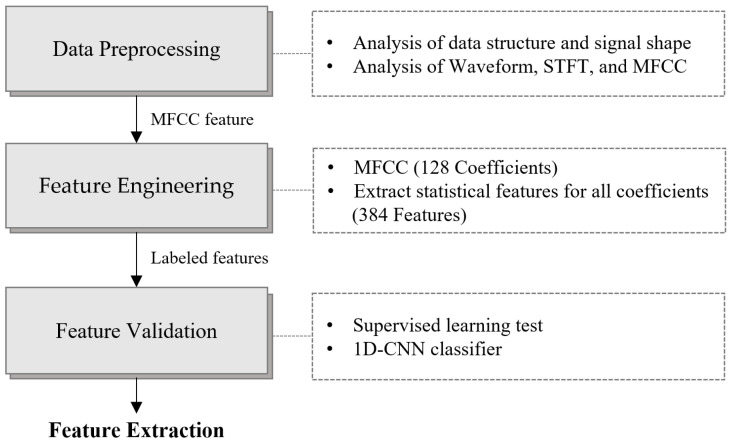
Feature extraction and validation procedure of the MIMII and forklift datasets.

This study uses the MIMII open dataset and forklift data directly acquired in a laboratory setting. The MIMII dataset contains acoustic signals with both normal and abnormal conditions collected from four types of equipment, namely fans, pumps, slide rails, and valves. The abnormal conditions in this dataset reflect actual fault conditions, such as wear, mechanical defects, and operational malfunctions. Forklift data were obtained via degradation experiments conducted in the laboratory, and noise from the MIMII dataset was added to simulate real operational environments. Previous studies have reported the use of two-dimensional (2D) CNNs with short-time Fourier transform (STFT) or MFCCs applied to the MIMII dataset [27,43]; however, this approach is computationally expensive and inefficient for practical applications. In this study, as the two sound datasets have different signal lengths, a feature extraction method that does not rely on signal length was applied. The extracted features were then validated using a 1D-CNN classifier. Figure 2 illustrates the data preprocessing and feature extraction processes used in this study. During the data preprocessing phase, the structure and waveform of the signals were analyzed, and the critical features were extracted using STFT and MFCCs. MFCC transformation yielded 128 coefficients, and an additional 384 statistical features were extracted from each coefficient. Finally, the extracted features were validated using a 1D-CNN classifier.

### 2.1. MIMII Dataset

**Figure 3:**
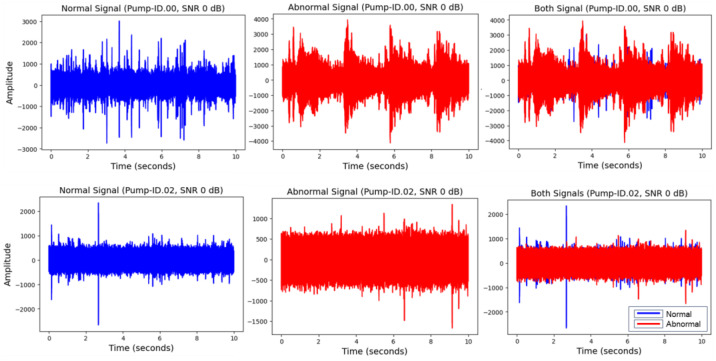
Waveforms obtained from pump ID.00 and .02 under normal and abnormal conditions.

The MIMII open dataset contains acoustic signals representing both normal and abnormal operating conditions. The MIMII dataset contains 16-kHz audio signals measured in 10 s segments for each condition, with a total of 16 data samples publicly available, including four model IDs for each machine [1]. Various anomalous sounds, such as contamination, rail damage, misalignment, and leakage, are present in the fault data, with a total of 18,019 normal and fault data samples (Table 1). Additionally, background noise recorded in actual factory settings was mixed with the target machine sounds. During the noise-mixing process, the signal-to-noise ratios (SNRs) were set to 6, 0, and −6 dB for each signal. To achieve this, the average power (a) of each machine model was calculated, and the power ($b_{j}$) of a randomly selected background noise segment (j) of each segment (i) was adjusted to satisfy $\gamma =10log10(a/b_{j})$. The adjusted noise signal was added to the target machine signal to generate noise-mixed data. Figure 3 illustrates the waveforms of the normal and abnormal conditions over approximately 10 s at an SNR of 0 dB for the pump (model ID.00 and .02) signals. The failure modes of abnormal conditions for each piece of equipment included in the MIMII dataset are summarized in Table 2. Additionally, the causes and signal characteristics for each failure mode are also summarized.

### 2.2. Forklift Dataset

**Figure 4:**
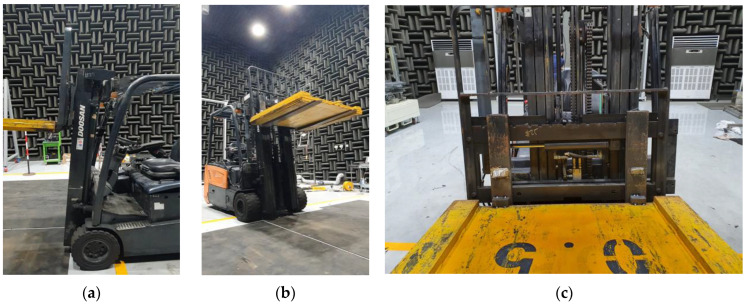
Acquiring front-end failure data of a forklift in an anechoic room: (**a**) left-side, (**b**) isometric, and (**c**) front views.

**Figure 5:**
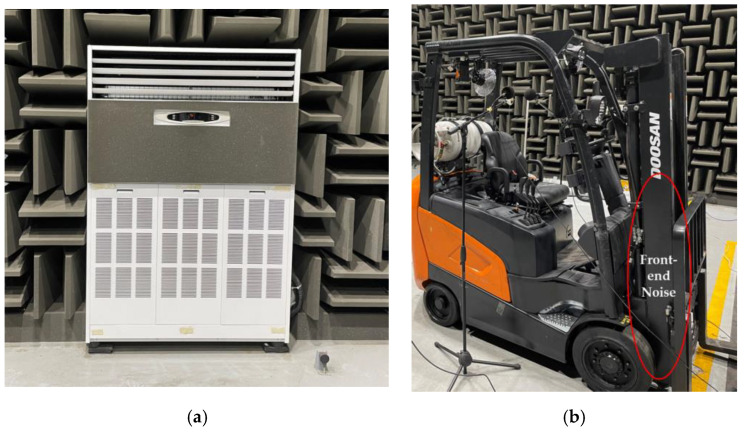
Acquiring background noise data in an anechoic room: (**a**) HVAC and (**b**) internal-combustion forklift noise.

An electric forklift was used in these experiments, with significant faults occurring in the front-end structure that lifts heavy loads. As shown in Figure 4, the experiments were conducted in a laboratory environment in which the forklift repeatedly lifted and lowered a 1500 kg off-centered load at a constant speed every 20 s for 5 h while remaining stationary. Audio signals were sampled at 51.2 kHz and divided into 20 s segments, creating condition segments. A microphone was placed at the operator’s position to record the noise generated from the front end (Figure 5). Acceleration tests were repeated until a fault occurred, during which the noise from the shaking forklift and its structures was measured. Additionally, the noise from the heating, ventilation, and air conditioning (HVAC) systems and engine-powered forklifts commonly used in factories and warehouses was artificially mixed as background noise. This background noise was mixed with the target signal at SNRs of 6, 0, and −6 dB, similar to the case of the MIMII dataset. Table 3 summarizes the structure of the forklift dataset, which includes a total of 5400 data samples, categorized according to the SNR and various types of background noise.

### 2.3. Exploratory Data Analysis and Feature Engineering

**Figure 6:**
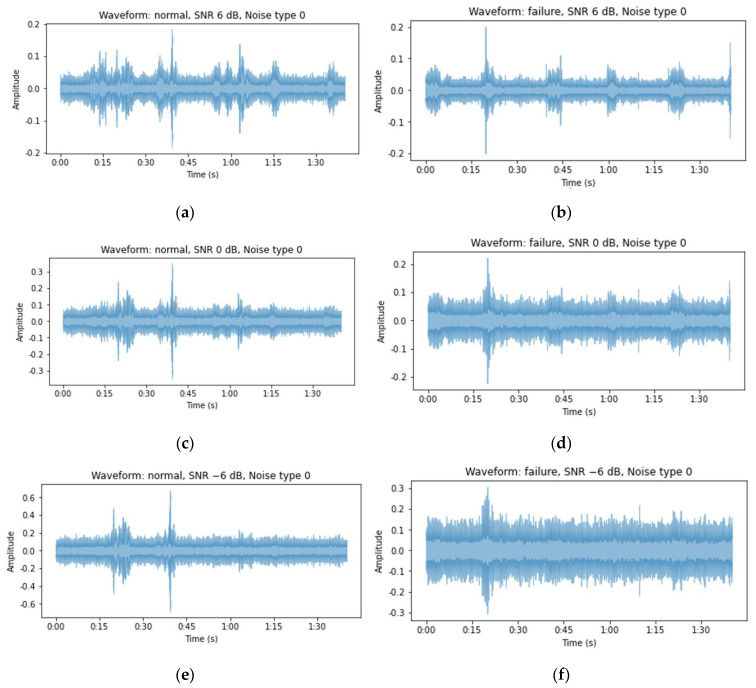
Visual examples of the forklift audio waveforms: (**a**) normal SNR: 6 dB, (**b**) failure SNR: 6 dB, (**c**) normal SNR: 0 dB, (**d**) failure SNR: 0 dB, (**e**) normal SNR: −6 dB, and (**f**) failure SNR: −6 dB.

STFT [44] and MFCCs [45] were used to analyze the time series of the acoustic signal and observe its features. First, the STFT applies Fourier transforms to vibration or sound signals and represents the results in the time–frequency domain, allowing the analysis of frequency changes over time in a 2D format. Converting the STFT results to a logarithmic scale allows the log spectrogram to preserve the magnitudes of the frequency components, enabling the observation of subtle signal changes [45]. When STFT was applied to the 10 s data from the MIMII dataset, 320,000 1D data samples were transformed into 513 frequency components and time frames each (513 × 513). In contrast, when STFT was applied to the 20 s data from the forklift dataset, the 320,000 1D data points were transformed into 513 frequency components and 626 time frames (513 × 626). The sampling rates of the MIMII and forklift datasets differed; therefore, the forklift dataset was down-sampled from 51.2 kHz to 16 kHz to match the sampling rate of the MIMII dataset. This process allowed the two datasets to be compared. Figure 6 displays the waveforms of the normal and fault states obtained from the forklift dataset over 100 s in a background-noise-type 0 environment. The surrounding noise was observed to increase, and the peak noise expected to be generated from the front-end operation of the forklift was masked by the background noise.

**Figure 7:**
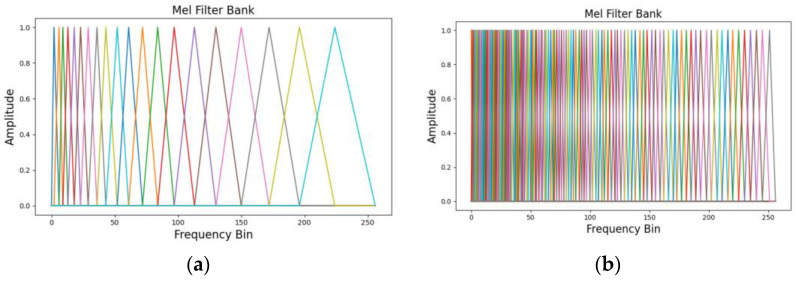
Visual examples of the Mel filter bank: the numbers of coefficients are (**a**) 20 and (**b**) 128.

The MFCCs were obtained by applying a Mel filter bank to the output of STFT and performing a logarithmic transformation. Subsequently, a discrete cosine transform was applied to the transformed data to convert the frequency components into the time domain. The extracted MFCCs represent the characteristics of the sound, reducing the dimensionality of the sound signal and emphasizing the signal features [45]. Such studies [46,47] demonstrate that using Mel filter banks and Mel frequency filters for fault diagnosis in rotating machinery is effective, with the Mel scale’s heightened sensitivity to low-frequency bands proving valuable in detecting equipment faults. In this study, MFCC transformation was applied by setting the number of MFCCs to 128 based on the STFT results (Figure 7). For STFT transformation, the window length was set to 1024, and the overlap window was set to 512. The 20 s data obtained from the forklift dataset were reduced in dimensionality from 320,000 to 128 × 626 via MFCC transformation, whereas the dimensionality of the 10 s data from the MIMII dataset was transformed to 128 × 513. Consequently, the MFCC results, which have fewer dimensions than the STFT results, were used as the critical features. Ultimately, each condition segment in the dataset had 384 feature vectors. This method allows for the generation of feature vectors that reflect the same sound-based physical characteristics, even if the segment lengths of the sound signals differ.

**Figure 8:**
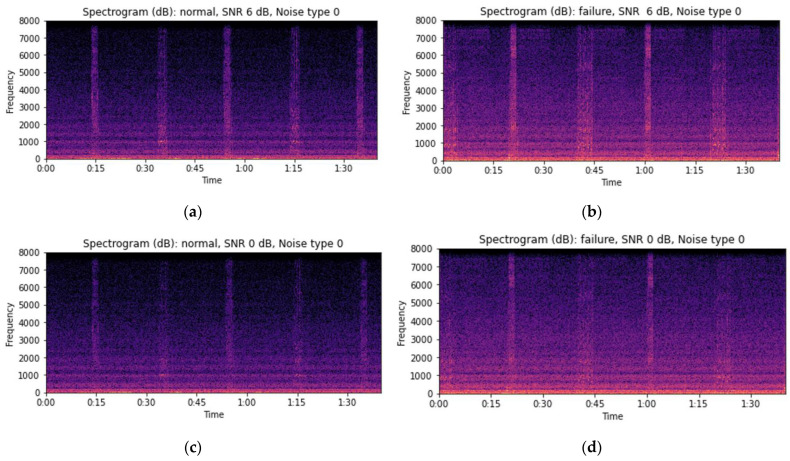

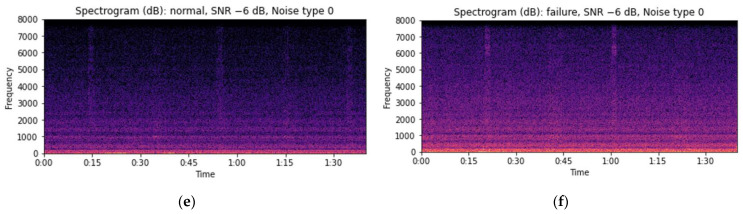
Visual examples of the forklift audio log spectrogram (STFT): (**a**) normal SNR: 6 dB, (**b**) failure SNR: 6 dB, (**c**) normal SNR: 0 dB, (**d**) failure SNR: 0 dB, (**e**) normal SNR: −6 dB, and (**f**) failure SNR: −6 dB.

**Figure 9:**
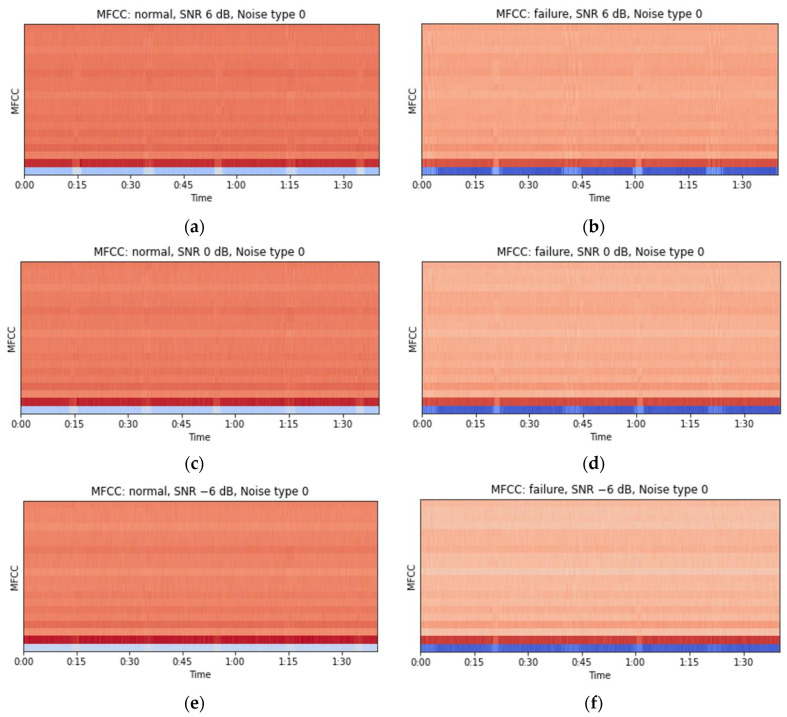
Visual examples of the forklift audio MFCC results obtained using 20 coefficients: (**a**) normal SNR: 6 dB, (**b**) failure SNR: 6 dB, (**c**) normal SNR: 0 dB, (**d**) failure SNR: 0 dB, (**e**) normal SNR: −6 dB, and (**f**) failure SNR: −6 dB.

The STFT spectrogram with a log scale applied and the MFCC analysis results reveal that changes in the signal can be identified at the expected moments of the front-end lifting operation of the forklift even as the background noise increases (Figure 8 and Figure 9). Following this, statistical features such as the mean, maximum, and minimum were extracted from each MFCC to reduce the dimensionality of the data. To simplify visualization, the number of MFCCs was reduced to 20 (Figure 9).

### 2.4. Feature Validation via Supervised Learning

**Figure 10:**
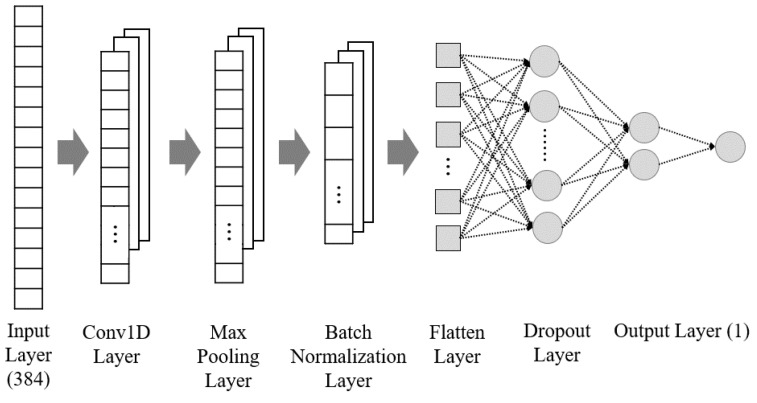
Structure of the 1D-CNN classifier from the input to the output.

To validate the extracted features, a fault diagnosis classifier based on a 1D-CNN was constructed, and supervised learning was conducted. The 1D-CNN, suitable for the classification of 1D data, consists of convolutional, pooling, and fully connected layers [48,49]. The ReLU activation function was applied to the results of the convolution operations to introduce nonlinearity. Max pooling was used to reduce the size of the feature map by selecting the maximum value from each region. Subsequently, a fully connected layer was employed for the final classification task, and the difference between the actual class labels and the predicted values was minimized using the cross entropy loss function. Additionally, batch normalization and dropout were incorporated to prevent overfitting. Finally, after connecting the flattened and fully connected layers, the sigmoid activation function was applied to perform binary classification of the normal and fault conditions. Figure 10 illustrates the overall layered structure of the 1D-CNN classifier. To address the class imbalance issue, the fault data in the MIMII dataset were down-sampled. Furthermore, 80% of the normal data in the forklift dataset were used for training, and the remaining 20% were used for performance evaluation. A total of 48 classifiers were created and evaluated based on the machine, ID, and SNR combinations present in the MIMII dataset, with an average accuracy of 96.79% for classification. For the forklift dataset, a total of six classifiers were created and evaluated based on SNR and background noise types, with an average accuracy of 99.69% for classification. These results validate the extracted features, indicating the applicability of these features to unsupervised learning.

## 3. Unsupervised Learning Using Variational Autoencoder (VAE)

**Figure 11:**
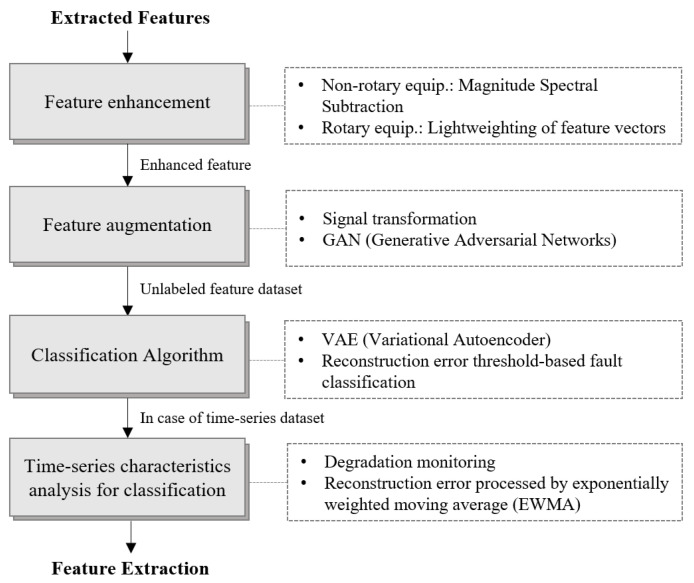
Fault diagnosis procedure using a VAE.

The features extracted in the previous section were enhanced and augmented, followed by training a VAE to conduct unsupervised learning-based fault diagnosis. Specifically, for the forklift dataset, which includes time-series features, we proposed a method for monitoring degradation states and improving performance by combining a VAE with EWMA. Figure 11 summarizes the fault diagnosis process, with each step structured as follows:

1. Feature enhancement: For nonrotating equipment, the magnitude spectral subtraction (MSS) technique was applied, and the feature vectors were optimized.
2. Feature augmentation: Signal transformation and GANs were used to expand and augment the existing features, improving learning performance.
3. Classification using a VAE: The new data were compared with the data corresponding to the learned normal state to determine whether the data represented a normal or fault condition.
4. Time-series data analysis: Degradation was monitored using reconstruction errors, and gradual degradation and faults were detected by analyzing these reconstruction errors using EWMA.

This approach allows for the consideration of both the static and dynamic features of acoustic signals.

### 3.1. VAE-Based Fault Diagnosis Process

**Figure 12:**
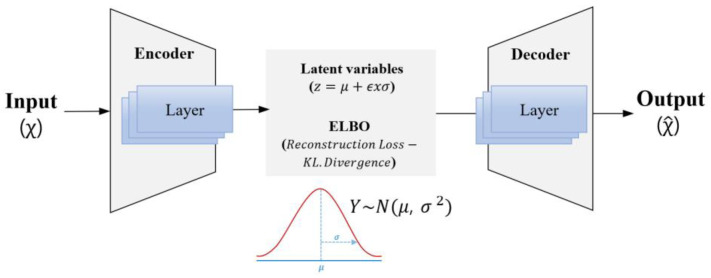
VAE architecture.

An autoencoder consists of an encoder, which converts unlabeled training data into a latent representation, and a decoder, which reconstructs the latent representation back into the output [50,51]. As the autoencoder reconstructs the input data, the output is referred to as the reconstruction, and mean square error (MSE) is typically used as the reconstruction loss during training. A VAE learns data features by transforming the input data into a latent space and reconstructing these data. Instead of compressing the input data into a specific latent space, a VAE learns by sampling from the probabilistic distribution model of the data. During the training process of a VAE, the latent variable (*z*) is sampled by calculating the mean (μ) and standard deviation (σ) of a given probability distribution and estimated using the expression z=μ+ϵxσ, where ϵ represents the noise sampled from the probability distribution. Via this process, the VAE learns the data distribution in the latent variable space. The decoder then accepts the latent variable as input and reconstructs the data (Figure 12). In this study, the latent variable was sampled under the assumption that the data follow a Gaussian normal distribution.

**Figure 13:**
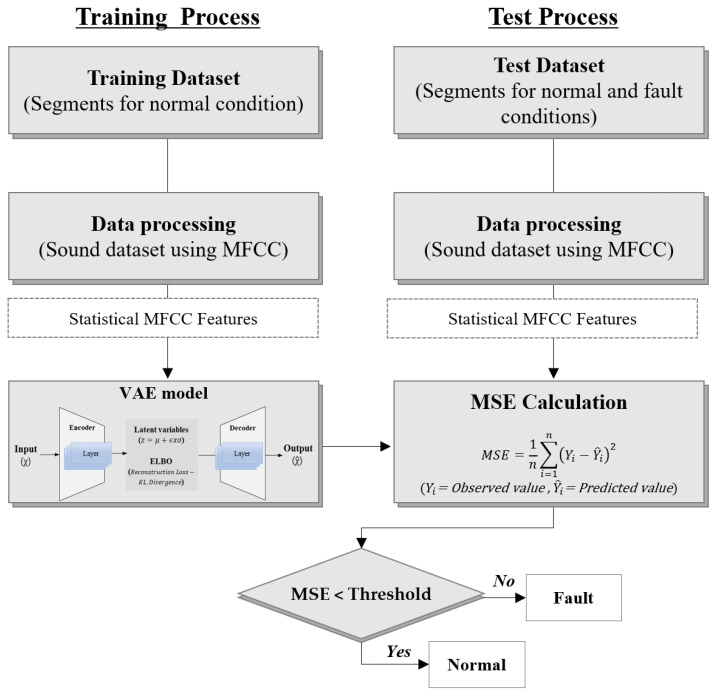
Fault diagnosis algorithm using the reconstruction error of the VAE.

The learning objective of a VAE is to maximize the evidence lower bound [52], defined by combining the reconstruction loss and the Kullback–Leibler (KL) divergence. The reconstruction loss represents the difference between the actual input and reconstructed data. KL divergence is a measure of the difference between the output distribution of the encoder and the Gaussian normal distribution and calculates the discrepancy between the latent variable distribution of the VAE and the normal distribution [53]. Under unsupervised fault diagnosis, when only normal state data are used for training the VAE, fault data typically yield a large reconstruction error [50,51,52,53]. This study implemented a fault diagnosis algorithm based on the VAE structure shown in Figure 12 by following the procedure depicted in Figure 13. The training data used the 384 generated feature vectors, and the VAE model was trained solely on normal state data to detect fault states. After setting a threshold based on the MSE distribution of the training data, the test data were reconstructed using the VAE. If the MSE of the test data was below the threshold, they were classified as normal. By contrast, if the MSE exceeded the threshold, the data were classified as faults.

### 3.2. Metrics and Dataset of VAE

The fault diagnosis accuracy of the VAE was evaluated using the area under the curve (AUC) score [54]. The AUC represents the area under the receiver operating characteristic curve and has a value between 0 and 1. In this study, the test data corresponding to the MIMII dataset were generated by randomly sampling the same number of normal data points as those of the fault data, and the remaining normal data were used for training. From the forklift dataset, in which the number of fault data points exceeds that of the normal data points, half the normal data were randomly sampled for training, and the remaining data were used for testing.

### 3.3. VAE-Based Fault Diagnosis Results

#### 3.3.1. Feature Enhancement

**Figure 14:**
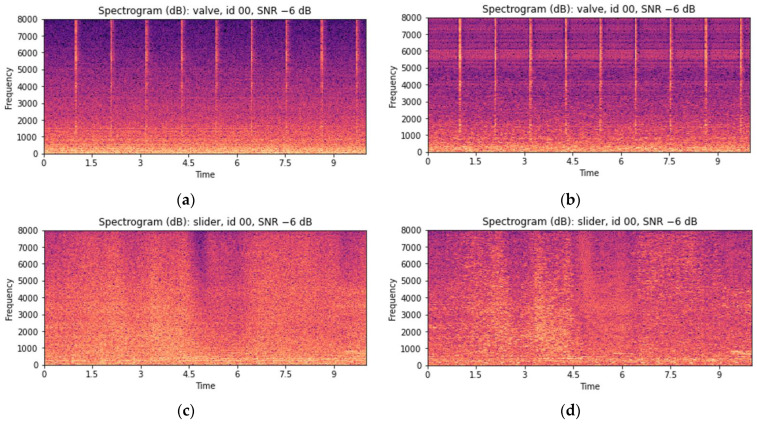
Visual examples depicting the MSS enhancement of valve and slide rail audio using log spectrograms (STFT): (**a**) before enhancement: valve with an SNR of −6 dB; (**b**) after enhancement: valve with an SNR of −6 dB; (**c**) before enhancement: slide rail with an SNR of −6 dB; and (**d**) after enhancement: slide rail with an SNR of −6 dB.

As valves and slide rails are nonrotating equipment, specific operations appear as peak signals in the spectrograms of raw signals. However, as the surrounding environmental noise increases, these peak signals become obscured in noise, making them difficult to observe. Figure 14a,c demonstrate this phenomenon. To address this issue, environmental noise was removed using MSS. MSS is commonly used to restore audio signals mixed with environmental noise or interference [54]. The noise-reduced STFT can be obtained by subtracting the STFT of the estimated noise signal from that of the original signal [14]. Because estimating a separate background noise spectrum using the MIMII dataset was difficult, the noise was estimated and removed directly from the original signal. The spectrogram changes corresponding to the valve and slide rail after applying MSS are shown in Figure 14b,d.

**Figure 15:**
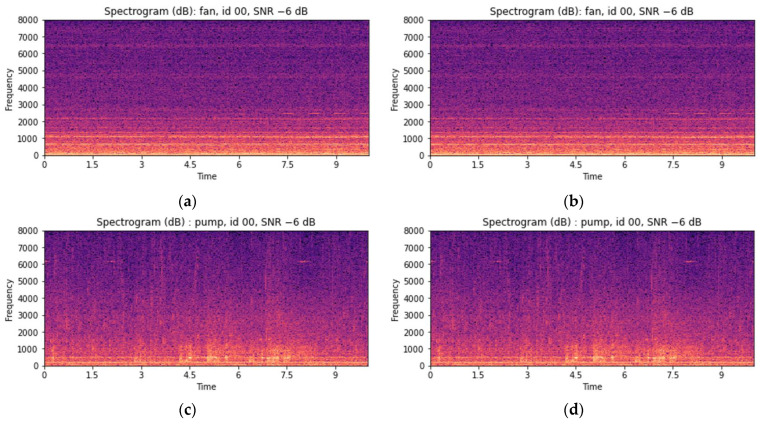
Visual examples depicting the MSS enhancement of valve and slide rail audio using log spectrograms (STFT): (**a**) before enhancement: fan with an SNR of −6 dB; (**b**) after enhancement: fan with an SNR of −6 dB; (**c**) before enhancement: pump with an SNR of −6 dB; and (**d**) after enhancement: pump with an SNR of −6 dB.

Rotating equipment such as fans and pumps, however, is affected by frequency fluctuations and vibrations owing to high-speed rotation. During the operation of rotating equipment, noise is generated owing to pressure changes, and instead of sharp peak signals, steady frequencies are observed in the corresponding spectrograms. Therefore, applying MSS to rotating equipment can distort the results based on these constant frequencies, as shown in Figure 15. This occurs when the noise spectrum is estimated based on the constant noise generated by the target equipment. Therefore, for rotating equipment, MSS was not applied, and the MFCC features were used as they were. Furthermore, as the constant frequencies generated by rotating equipment tend to have maximum and minimum values similar to the average values over time, only the average features were used to reduce the feature vector.

#### 3.3.2. Feature Augmentation

**Figure 16:**
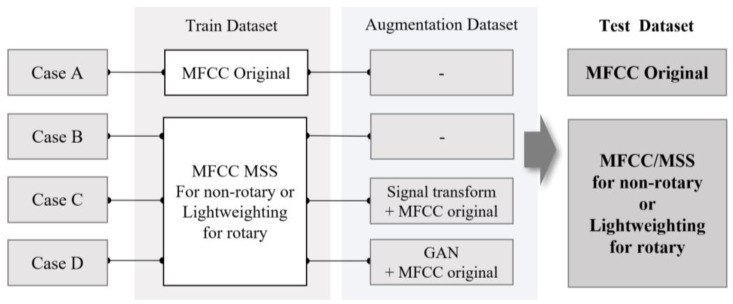
Combining the training dataset with the augmentation dataset and constructing the test dataset.

In this study, sound data were augmented using signal transformation techniques such as time stretching, time reversal, and polarity inversion. Time stretching is a technique that adjusts the playback speed of an audio signal while maintaining the pitch, increasing or decreasing the length of the signal [55]. Time reversal reverses the sequence of the signal, and polarity inversion transforms the sound signal by inverting the signal amplitude [56]. These techniques were used to double the normal data used for augmentation, and these data were then incorporated into the training data. Additionally, the extracted feature vectors were augmented using GANs, consisting of two networks: a generator, which generates fake data that resemble real data, and a discriminator, which distinguishes between the fake data generated by the generator and real data [57]. In this study, the training data were augmented to twice the size of the original normal data. Through MSS enhancement, feature optimization, augmentation of sound signal transformation, and GAN augmentation, data cases are constructed as shown in Figure 16.

#### 3.3.3. Analysis Results Obtained Using Nonrotating Equipment

**Figure 17:**
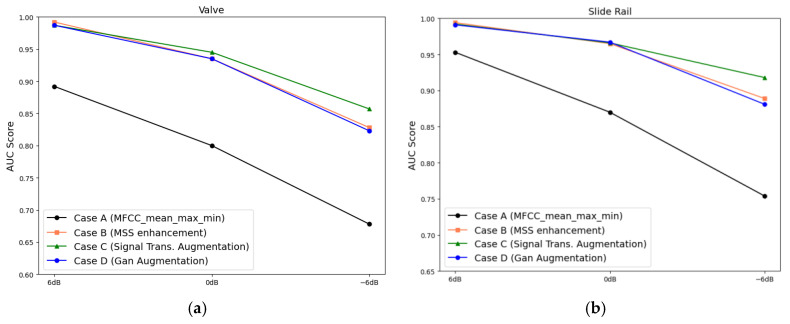
Comparing the AUC scores of each dataset for nonrotating equipment: (**a**) valve and (**b**) slide rail.

Faults of nonrotating equipment are diagnosed for each case depicted in Figure 16. The AUC scores corresponding to the valve and slide rail under SNR conditions of −6, 0, and 6 are shown in Figure 17a,b. In case A, the average AUC scores under the SNRs of 6, 0, and −6 dB were 0.9227, 0.8348, and 0.7164, respectively. In case B, the AUC scores improved to 0.9927, 0.9499, and 0.8586, and in case C, the AUC scores further improved to 0.9894, 0.9554, and 0.8876, respectively. In case D, similar results were obtained under SNRs of 6 and 0 dB; however, the AUC score decreased to 0.8517 under the SNR of −6 dB (Table 4). These results indicate that data augmentation using GANs is ineffective for data containing high noise levels.

#### 3.3.4. Analysis Results Obtained Using Rotating Equipment

**Figure 18:**
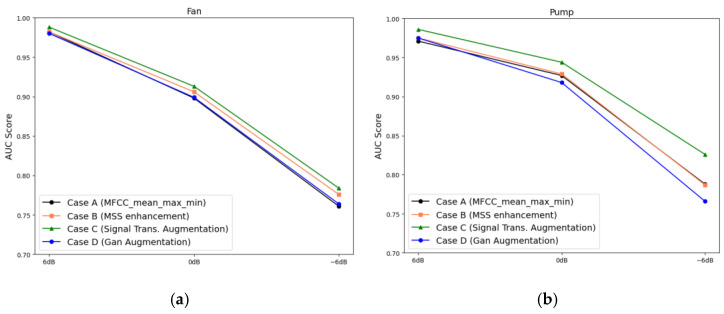
Comparing the AUC scores of each dataset for rotating equipment: (**a**) fan and (**b**) pump.

Faults in rotating equipment were diagnosed for each case. The prediction performances (AUC score) corresponding to a fan and pump under SNR conditions of –6, 0, and 6 dB are shown in Figure 18a,b. The average AUC scores in case A under SNRs of 6, 0, and −6 dB were 0.9769, 0.9125, and 0.7742, respectively. In case B, the AUC scores increased slightly to 0.9782, 0.9175, and 0.7816, and in case C, the AUC scores further increased to 0.9870, 0.9284, and 0.8052, respectively. However, in case D, the AUC scores decreased slightly under SNRs of 6 and 0 dB, and under an SNR of −6 dB, the AUC score decreased substantially to 0.7650, indicating the absence of the data augmentation effect. Table 5 lists the AUC values obtained in each case. These results also suggest that data augmentation using GANs is ineffective for datasets containing high noise levels.

#### 3.3.5. Forklift Analysis Results

**Figure 19:**
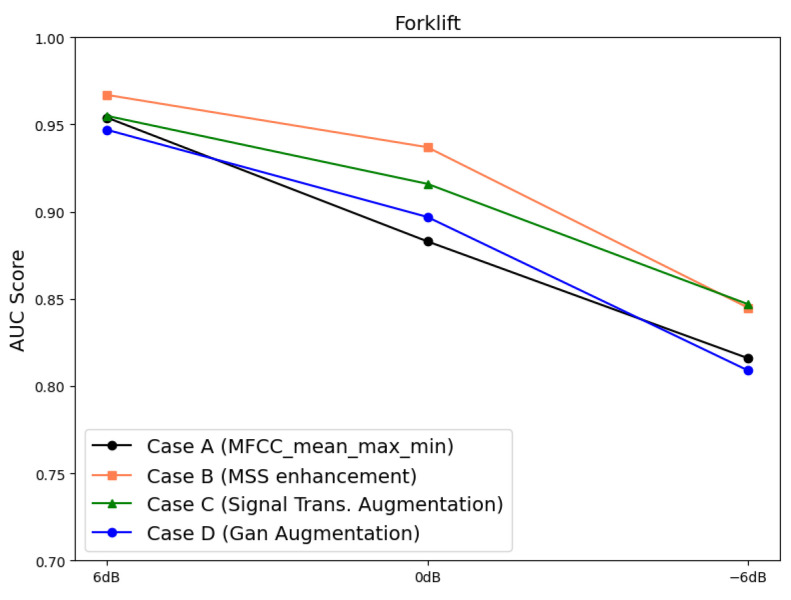
AUC score comparison across cases and SNR levels corresponding to the forklift.

As the front-end structure of the forklift represents nonrotating equipment, the same feature extraction and data augmentation techniques used for the valve and slide rail in the MIMII dataset were applied. The fault diagnosis results obtained using this front-end structure are displayed in Figure 19. In case A, the average AUC scores under SNRs of 6, 0, and −6 dB were 0.9544, 0.8827, and 0.8159, respectively. In case B, the AUC scores improved to 0.9667, 0.9366, and 0.8453, respectively. However, in case C, the AUC scores decreased to 0.9551, 0.9155, and 0.8469, respectively. In case D, as the level of environmental noise increased, the AUC score decreased, showing no significant effect (Table 6). This can be attributed to the smaller volume of training data than the volume of data in the MIMII dataset, overfitting the model when the augmented feature dataset was used and thereby deteriorating the performance.

### 3.4. VAE Analysis of Forklift Dataset Considering Time-Series Characteristics

**Figure 20:**
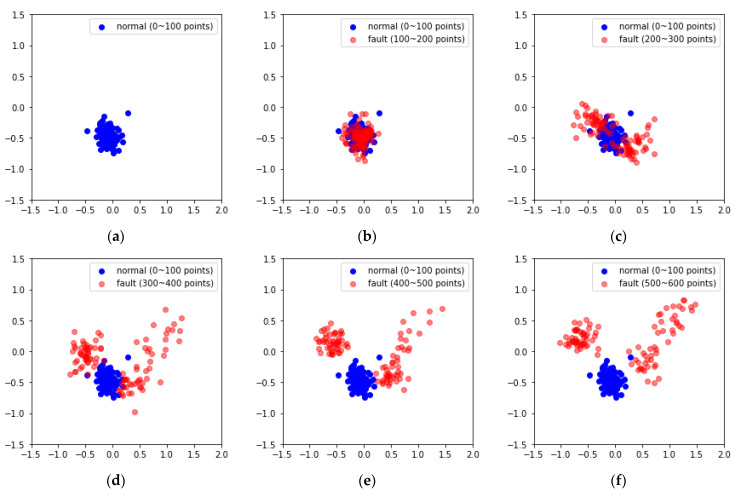

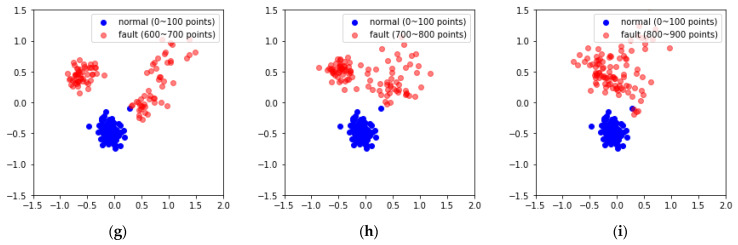
PCA visual examples using case 2, noise type 00, SNR = 6 dB: (**a**) 100 (**b**) 100–200, (**c**) 200–300, (**d**) 300–400, (**e**) 400–500, (**f**) 500–600, (**g**) 600–700, (**h**) 700–800, and (**i**) 800–900 data points.

**Figure 21:**
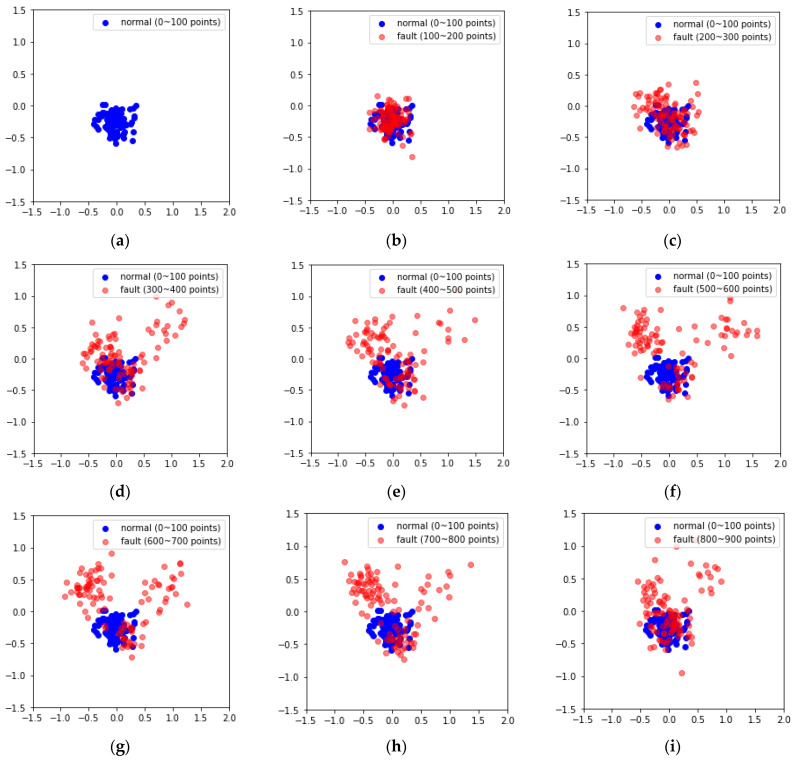
PCA visual examples using case 2, noise type 00, SNR = −6 dB: (**a**) 100, (**b**) 100–200, (**c**) 200–300, (**d**) 300–400, (**e**) 400–500, (**f**) 500–600, (**g**) 600–700, (**h**) 700–800, and (**i**) 800–900 data points.

The MIMII dataset consists of fixed segment data with arbitrary fault modes, which do not reflect changes over time. However, the data in the forklift dataset used in this study were collected over time, allowing for the reflection of such time-series characteristics. To account for this, the latent variables generated by the VAE were compressed into two principal components using PCA, and the changes over time were visually analyzed. PCA is a technique that reduces the dimensionality of data, generating new variables known as principal components and preserving the key information contained in the data [58]. The data points corresponding to principal components 1 and 2 in the forklift dataset used herein increased in distance from the normal data over time (Figure 20). Additionally, in the datasets containing high noise levels, some fault data points were found to overlap with normal data regions (Figure 21).

**Figure 22:**
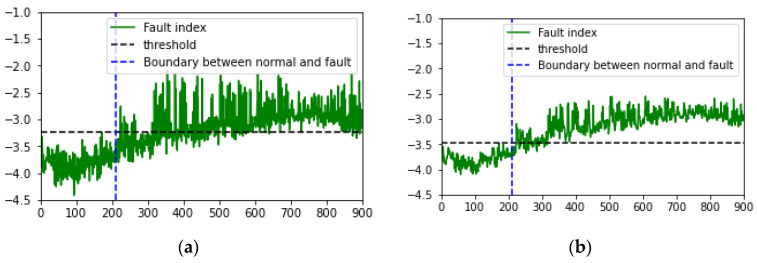
Visualized fault index (reconstruction error, MSE) under case 2, noise type 00, SNR = 6 dB: (**a**) before and (**b**) after EWMA (alpha = 0.5 and three points).

**Figure 23:**
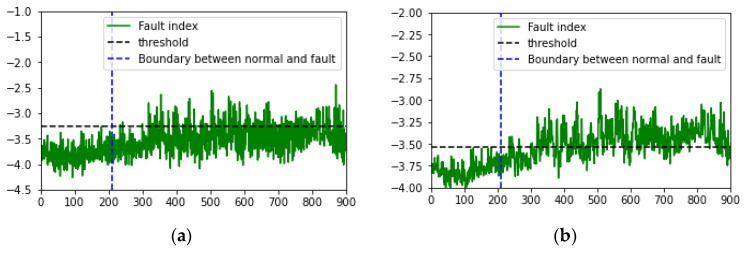
Visualized fault index (reconstruction error, MSE) under case 2, noise type 00, SNR of −6 dB: (**a**) before and (**b**) after EWMA (alpha = 0.5 and three points).

The MSE derived from the forklift dataset using the VAE was set as the fault index, and the analysis over time revealed that the fault index gradually increased. This indicates early signs of failure that appear as the forklift degrades before a fault occurs. However, as environmental noise increased, the fault index became increasingly noisy, with the increase in variability and frequency of the peaks. To minimize the impact of past data during fault index analysis and simultaneously reduce noise and outliers, EWMA was applied [59,60]. The moving average method exponentially decays the influence of past data. As shown in Figure 22 and Figure 23, applying the moving average simplified the distinction between normal and fault data based on the fault index and the threshold.

**Figure 24:**
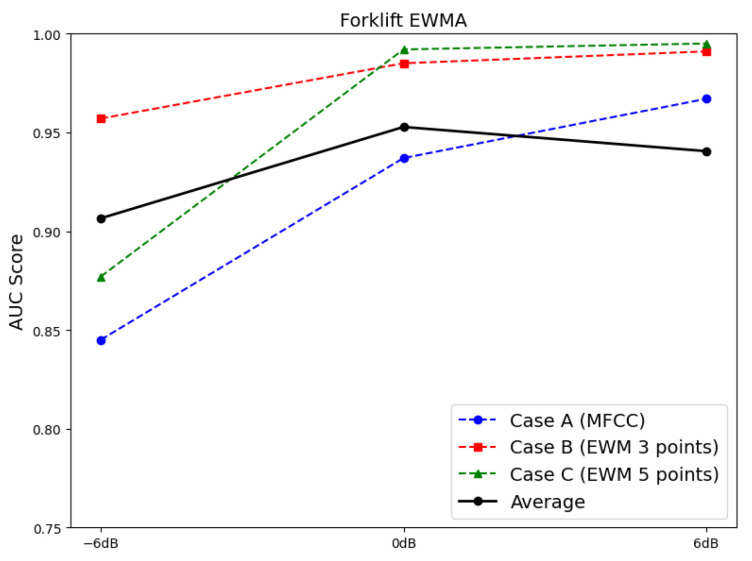
Comparison between the AUC scores for diagnosing faults in a forklift using the EWMA-applied MSE criteria.

In case B, the window size was set to three (60 s) and five points (100 s), and faults were diagnosed based on the MSE calculated using EWMA. Figure 24 compares the AUC scores under SNRs of –6, 0, and 6 dB. The results reveal that the AUC score increased from an average of 0.8453 at an SNR of −6 dB to 0.9567 and 0.9769 at SNRs of 0 and 6 dB, respectively.

## 4. Unsupervised Learning Using Domain Adaptation

**Figure 25:**
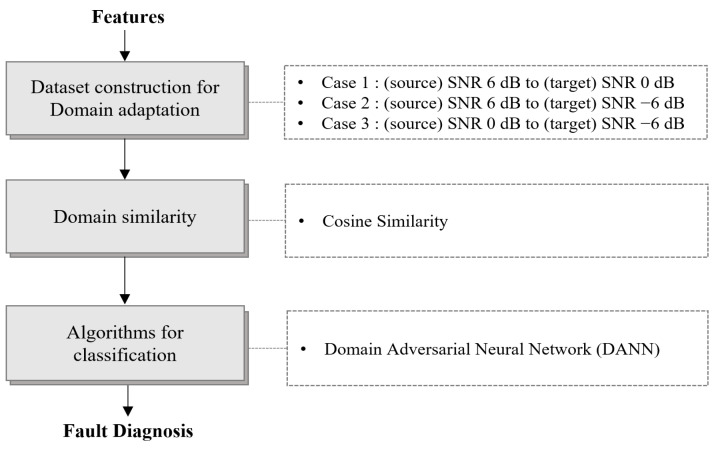
Fault diagnosis procedure using a DANN.

Domain adaptation is a technique used to maintain or improve model performance when a distribution difference exists between the source and target data [19]. Such distribution differences typically occur when data are collected from different domain environments. Domain adaptation compensates for these interdomain differences, enabling the trained model to adapt to out-of-domain data. In fault diagnosis, labels for both normal and fault data can be easily obtained in source environments such as laboratories. However, in target environments in which equipment is actually under operation, inducing artificial faults is difficult, and obtaining sufficient fault data is considerably challenging. In this section, we applied domain adaptation techniques by considering the differences in environmental noise as interdomain differences. The MIMII dataset with an SNR of 6 and 0 dB was used as the source data acquired in the laboratory, and supervised learning was implemented. Subsequently, data with SNRs of 0 and −6 dB were set as the target data, and domain adaptation was applied. Figure 25 illustrates the fault diagnosis process based on a DANN and explains the noise levels and composition between domains in the MIMII dataset.

### 4.1. Domain-Adversarial Neural Network (DANN)

**Figure 26:**
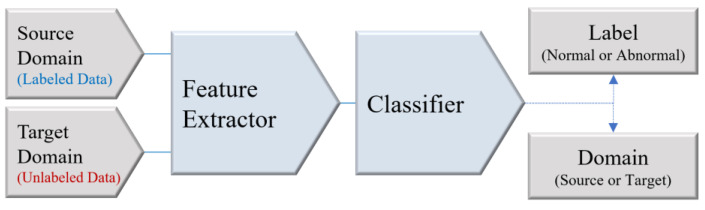
Architecture of the DANN.

The DANN is a neural network technique used to maintain or improve model performance when a distribution difference exists between the source and target data [61]. The concept of DANN involves the combined use of a domain classifier, feature extractor, and label classifier (Figure 26). The feature extractor extracts common features from the input data. The label classifier classifies based on labels or categories in the source domain using the extracted features. The domain classifier receives the output from the feature extractor and determines the domain to which the data belong. A critical component of a DANN is the GRL [61,62], used to ensure that the feature extractor learns domain-invariant features by removing the differences between the source and target domains. The GRL deceives the domain classifier such that the feature extractor cannot distinguish between the source and target domains [63]. The DANN training procedure involves two steps: (1) the feature extractor is trained to perform classification tasks on the source data and (2) the feature extractor is trained on both the source and target data for domain classification tasks. During this process, the GRL inverts the gradient sign during the backpropagation of the domain classification, enabling the learning of a common feature extractor. In this study, the 1D-CNN classifier described in Section 2.4 was used as the feature extractor, and both the label and domain classifiers were composed of fully connected layers.

### 4.2. Metrics and Dataset Corresponding to DANN-Based Fault Diagnosis

**Figure 27:**
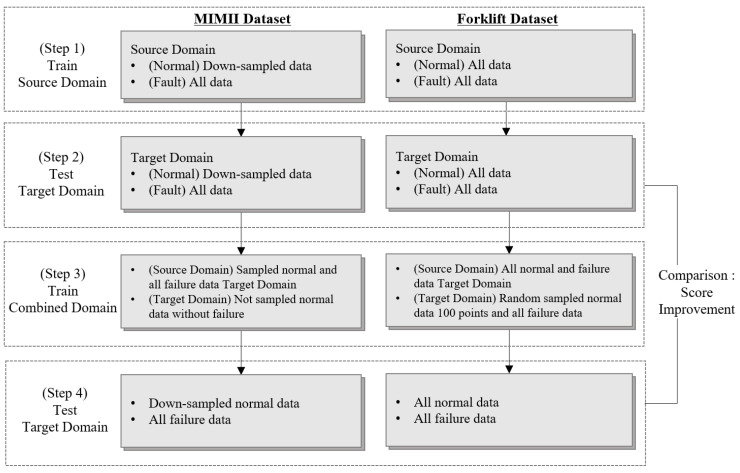
Domain adaptation procedure using the MIMII and forklift datasets.

**Figure 28:**
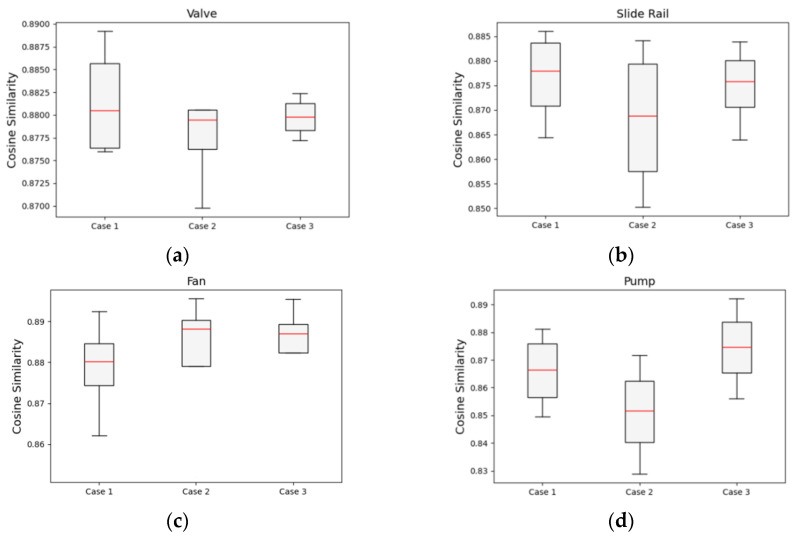
Cosine similarity results obtained from the MIMII dataset: (**a**) valve, (**b**) slide rail, (**c**) fan, and (**d**) pump.

During DANN-based fault diagnosis, the difference in environmental noise between the MIMII and forklift datasets was considered the domain difference. The MIMII dataset contains more normal data than fault data; therefore, the source dataset was down-sampled for training. For domain adaptation training, only normal data from the target dataset were used alongside the source data. The binary classification accuracy between the normal and fault states in the down-sampled target data was set as the evaluation metric. The training process for fault classification consists of four steps, as shown in Figure 27. In step 1, the model is trained using only the source data, and in step 2, the trained model is preliminarily evaluated using unseen target data. In step 3, the domain classifier is trained with the GRL by combining the target training and source domain data. Finally, in step 4, faults are diagnosed in the target domain, and the performance metrics are compared with the results obtained in step 2. As the forklift dataset contains more fault data than normal data, 100 normal data points were randomly sampled and used for training along with the source data (Figure 28). Owing to the imbalance in the test dataset, the F1-score [62] was used as the performance evaluation metric owing to the effectiveness of using this metric in cases in which one class contains a substantially larger or smaller number of samples than the other.

### 4.3. Domain Similarity of the Dataset

To verify the domain shift between the source and target data, cosine similarity between the domains was calculated. Cosine similarity is a measure of the closeness with which two vectors align in a direction, with values in the range of −1 to 1, where values close to 1 and −1 indicate high and low similarities, respectively [63]. The cosine similarity between the source and target in the MIMII dataset was an average of 0.8745, with a maximum of 0.8956 and a minimum of 0.8288 (Figure 28). This indicates a high level of similarity despite different SNR conditions. However, as the difference between SNR levels increased, such as in case 2, the cosine similarity values decreased. This suggests the need for domain adaptation techniques to reduce interdomain differences and improve model performance.

**Figure 29:**
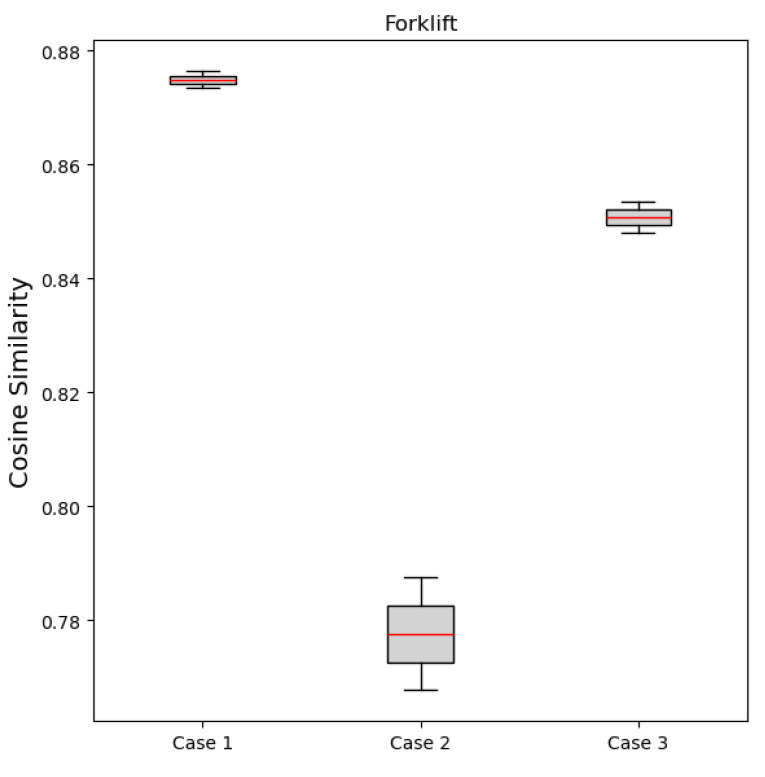
Cosine similarity results obtained using the forklift dataset.

Similarly, the cosine similarity between the two domains was calculated using the forklift dataset. For the six scenarios present in the forklift dataset, the cosine similarity between the source and target was an average of 0.8344, with a maximum of 0.8764 and a minimum of 0.7677 (Figure 29). This indicates a high level of similarity despite different SNRs. However, the cosine similarity obtained using the forklift dataset was lower than that obtained using the MIMII dataset as the difference between SNR levels increased, particularly in case 2.

### 4.4. DANN-Based Fault Diagnosis Results

#### 4.4.1. Non-Rotary Equipment Results

**Figure 30:**
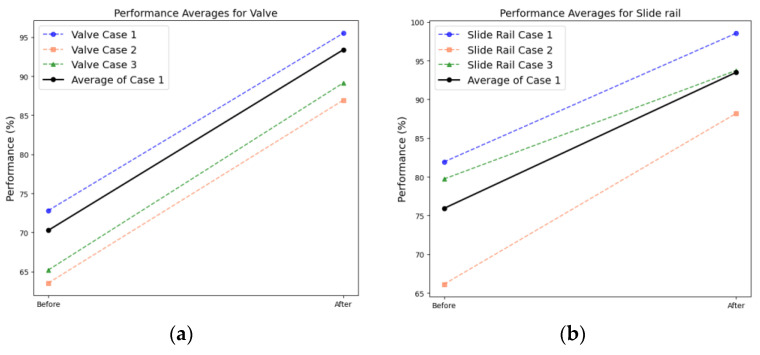
Comparison between the F1-scores corresponding to the DANN-based fault diagnosis results obtained from MIMII nonrotating equipment: (**a**) valve and (**b**) slide rail.

**Figure 31:**
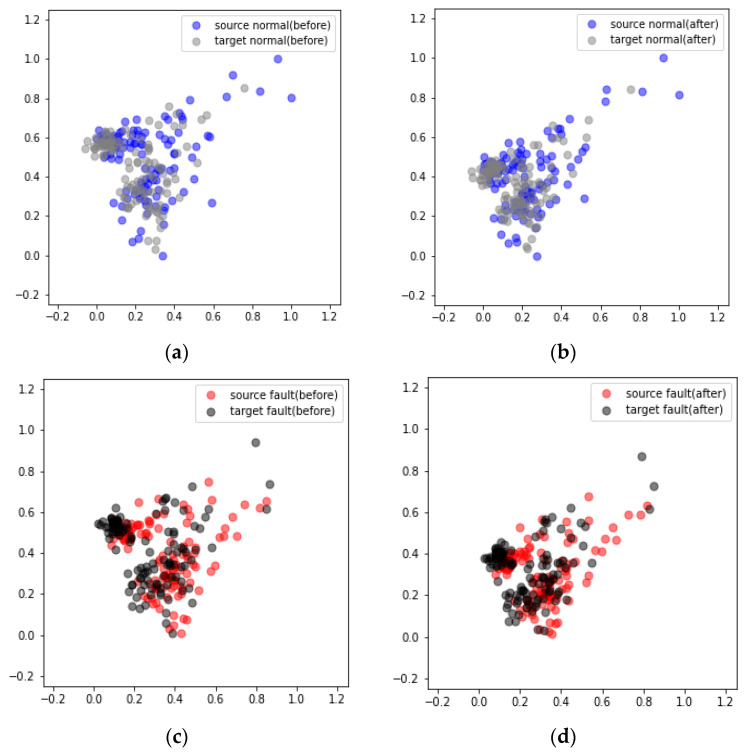
Visualization of the domain adaptation results obtained from the valve using PCA under case 3 and machine ID 00: (**a**) Before and (**b**) after the adaptation of normal data. (**c**) Before and (**d**) after the adaptation of failure data.

**Figure 32:**
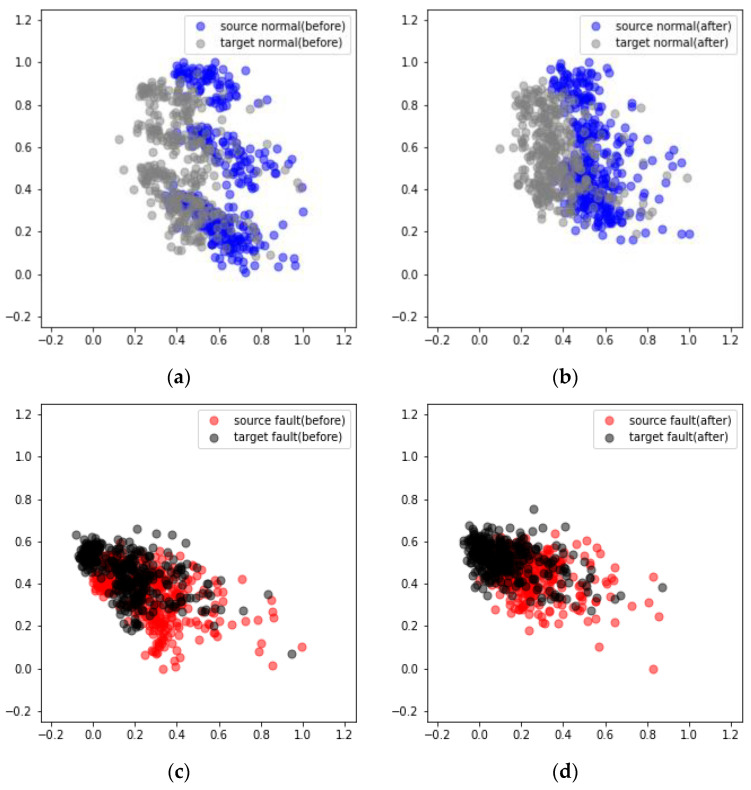
Visualization of the domain adaptation results obtained from the slide rail using PCA under case 3 and machine ID 00: (**a**) Before and (**b**) after the adaptation of normal data. (**c**) Before and (**d**) after the adaptation of failure data.

Figure 30 presents the results obtained from the DANN-based fault diagnosis on each piece of nonrotating equipment in the MIMII dataset. The diagnosis results reveal that in cases 1, 2, and 3 the average scores increased from 77.37% to 97.03%, 64.84% to 87.57%, and 72.46% to 91.45%, respectively (Table 7). However, the performance improvement in case 2 after applying the DANN was relatively lower than those in cases 1 and 3. The performance of domain adaptation decreased as the environmental differences between the source and target domains increased. Additionally, the values passing through the feature extractor before and after domain adaptation were compared using PCA. The comparison results reveal that after domain adaptation, the data points in the target domain formed a distribution similar to that of the source domain (Figure 31 and Figure 32).

#### 4.4.2. Analysis Results Obtained from Rotating Equipment

**Figure 33:**
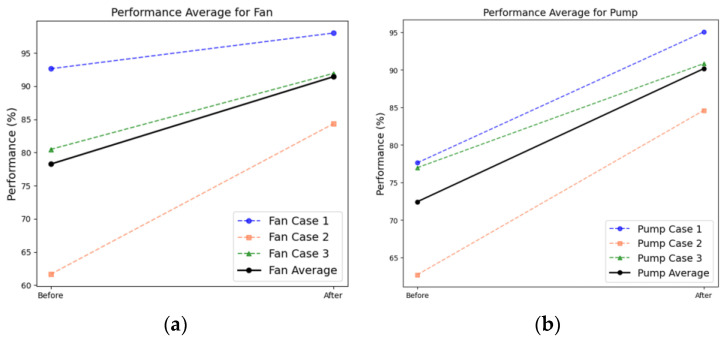
Comparison of the F1-scores corresponding to DANN-based fault diagnosis results obtained using MIMII rotating equipment: (**a**) fan and (**b**) pump.

**Figure 34:**
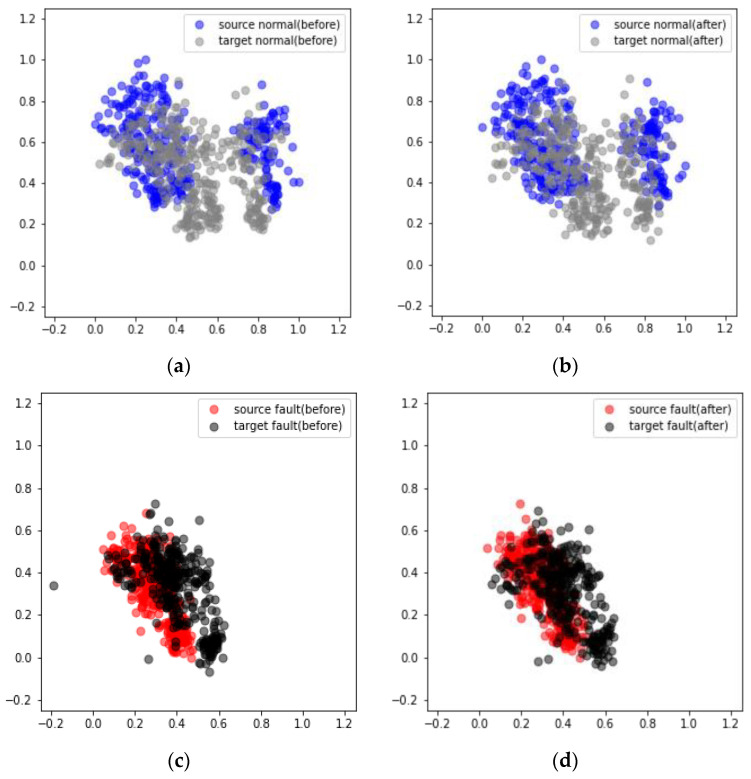
Visualization of the domain adaptation results obtained from a fan using PCA under case 3 and machine ID 04: (**a**) Before and (**b**) after the adaptation of normal data. (**c**) Before and (**d**) after the adaptation of failure data.

**Figure 35:**
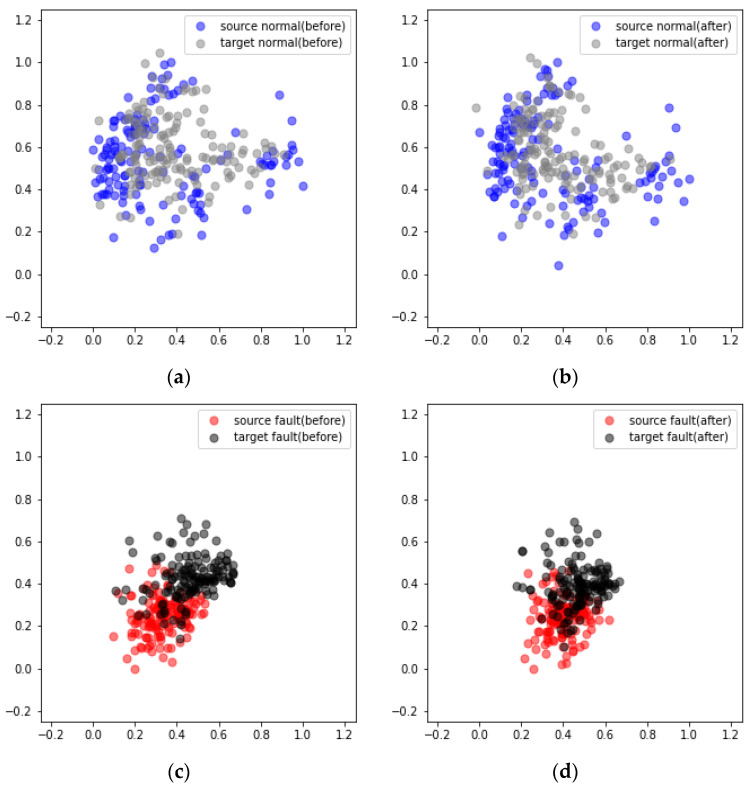
Visualization of the domain adaptation results obtained from a pump using PCA under case 3 and machine ID 00: (**a**) Before and (**b**) after the adaptation of normal data. (**c**) Before and (**d**) after the adaptation of failure data.

Figure 33 displays the results obtained from applying domain adaptation to rotating equipment represented in the MIMII dataset. The diagnosis results indicate that in cases 1, 2, and 3, the average scores increased from 85.12% to 96.52%, 62.18% to 84.47%, and 78.72% to 91.38%, respectively (Table 8). This trend is similar to that observed in the case of nonrotating equipment. The comparison of the results after applying domain adaptation using PCA reveals that the data points in the target domain formed a distribution similar to that of the source domain (Figure 34 and Figure 35).

#### 4.4.3. Analysis Results Obtained from a Forklift

**Figure 36:**
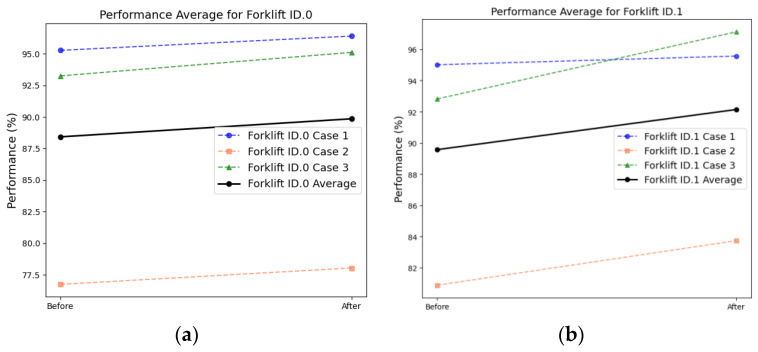
Comparison of the F1-scores corresponding to the DANN-based fault diagnosis results obtained from a forklift: equipment (**a**) ID.0 and (**b**) ID.1.

**Figure 37:**
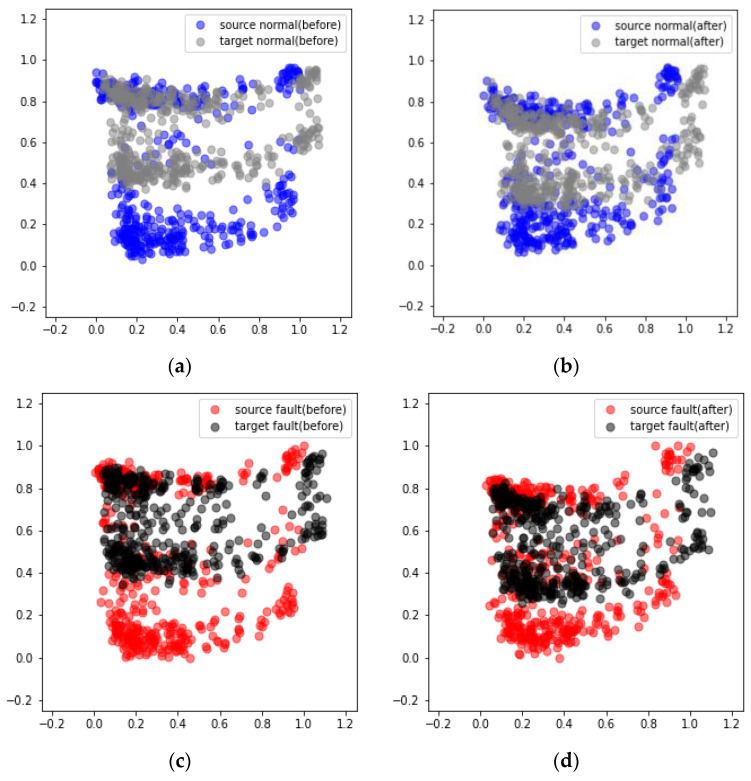
Visualization of the domain adaptation results obtained from a forklift using PCA under case 3 and noise type 00: (**a**) Before and (**b**) after the adaptation of normal data. (**c**) Before and (**d**) after the adaptation of failure data.

Figure 36 presents the results of the DANN-based fault diagnosis obtained using the forklift dataset. In cases 1, 2, and 3, the average scores increased from 95.14% to 95.99%, 78.82% to 80.89%, and 93.04% to 96.12%, respectively (Table 9). This confirms that the trend of domain adaptation is similar, regardless of the characteristics of the rotating equipment. A comparison of the results before and after domain adaptation using PCA revealed that the data points in the target domain formed a distribution similar to those of the source domain (Figure 37).

### 4.5. Discussion Based on the Results

This study proposed a fault diagnosis method using the sound dataset obtained from industrial equipment, including environmental noise, by the combined use of an unsupervised learning-based VAE and a DANN. The VAE and DANN algorithms were applied and the obtained results were compared using sound datasets obtained from nonrotating and rotating equipment, i.e., the MIMII and forklift sound datasets. A VAE, a probabilistic generative model based on an autoencoder, learns using only normal state data and uses the reconstruction error as a fault index to classify faults. DANN is a deep learning architecture for transductive transfer learning, reflecting domain shifts between the source and target domains via domain adaptation. This approach allows faults to be diagnosed by training within the source domain, such as a laboratory environment or a domain with low noise. DANN learning can then be applied to the target domain, representing the actual operational environment. For preprocessing sound data, the obtained waveforms, STFT, and MFCCs were analyzed to reduce data dimensionality and extract statistical features (using the MFCCs), capturing the physical characteristics of the signal. Using the statistical features of the acoustic signal obtained from the MFCCs, a feature vector shape that can be applied regardless of the length of the audio signal was extracted. Based on the characteristics of rotating equipment, MSS and feature optimization were used for feature enhancement, whereas audio signal transformation and GAN-based data augmentation were applied to improve performance and prevent overfitting. Faults were diagnosed by constructing cases (A–D) based on feature enhancement and augmentation configuration. The fault diagnosis results revealed that the nonrotating equipment represented in the MIMII dataset, i.e., case C, which applied both feature enhancement and signal transformation augmentation, yielded the highest AUC score. The forklift dataset, i.e., case B, which applied only feature enhancement, achieved the highest AUC score. These results suggest that the smaller volume of training data corresponding to the forklift dataset than that corresponding to the MIMII dataset caused overfitting when augmentation was implemented, deteriorating the performance based on the augmented feature dataset.

Furthermore, this study applied a time-series fault dataset representing the front end of a forklift, incorporating the time-series characteristics of the equipment, to monitor the degradation state of the forklift. By examining the latent variables encoded by the VAE using PCA in a 2D scatter plot over time, a clear boundary between the principal components of the normal and fault states was observed. However, as environmental noise increased, some overlap occurred between the principal components of the normal and fault states. To address this, the VAE reconstruction error corresponding to the degradation state was set as the fault index to assess the degradation of the forklift. Additionally, EWMA was applied during fault index analysis to minimize the influence of past data while reducing noise and outliers. On reapplying fault diagnosis, the AUC score increased from 0.8453 to 0.9567 and 0.9769 under an SNR of −6 dB in case 2. These findings confirm that if only normal state data are used for training along with time-series characteristics, faults can be successfully diagnosed even using sound datasets containing substantial environmental noise.

## 5. Conclusions

Among the two unsupervised learning-based fault diagnosis methods, a VAE can generate classifiers using only normal state data. However, the characteristics of both nonrotating and rotating equipment must be considered when implementing improvements and augmentation techniques for the features. Additionally, accuracy tends to decrease as the environmental noise level increases. A DANN does not require additional data processing based on the characteristics of nonrotating and rotating equipment and can diagnose faults in the target domain using labels from the source domain, regardless of the environmental noise level. By combining these algorithms, highly robust fault diagnosis can be achieved by rapidly diagnosing potential failures using a DANN once the equipment is installed while considering the equipment and time-series characteristics using a VAE. Furthermore, a complementary approach can be established to observe equipment degradation and detect hidden failures using a VAE. However, detecting hidden failures and predicting the RUL based on the fault index will require further investigations in the future.

## Figures and Tables

**Table 1:** MIMII dataset configuration under SNRs of 6, 0, and −6 dB.

Machine Type	Model ID	Segments for Normal Conditions	Segments for Anomalous Conditions	Sum
Valve	0	991	119	1110
	2	708	120	828
	4	1000	120	1120
	6	992	120	1112
Slide rail	0	1068	356	1424
	2	1068	267	1335
	4	534	178	712
	6	534	89	623
Fan	0	1011	407	1418
	2	1016	359	1375
	4	1033	348	1381
	6	1015	361	1376
Pump	0	1006	143	1149
	2	1005	111	1116
	4	702	100	802
	6	1036	102	1138
Total		14,719	3300	18,019

**Table 3:** Forklift dataset configuration under SNRs of 6, 0, and −6 dB.

Machine Type	Background Noise Type	Segments for Normal Conditions	Segments for Failure Conditions	Sum
Forklift	0	211	689	900
	1	211	689	900
Total		422	1378	1800

**Table 2:** The causes and signal characteristics for failure mode of the MIMII dataset [1].

Machine Type	Failure Mode	Cause	Signal Characteristics
Valve	Leakage	Seal leakage, improper closure	Irregular noise, high-frequency sound
	Clogging	Debris accumulation, blockage	Pressure change, disrupted fluid flow
	Wear and tear	Long-term usage, surface erosion	Gradual noise increase, inconsistent sound
	Deformation	Structural deformation, physical damage	Abnormal vibration, misalignment noise
Slide rail	Increased friction	Lack of lubrication, dust accumulation	Squeaking noise, increased frictional sound
	Wear and tear	Repetitive motion, surface wear	Gradual noise increase, uneven sound
	Structural deformation	External impact, bending	Impaired movement, vibration
	Foreign object accumulation	Dust, debris accumulation	Obstructed movement, scraping sound
Fan	Rotational imbalance	Blade damage, dust accumulation	Increased vibration, irregular high-frequency noise
	Bearing damage	Bearing wear, excess friction	Unstable sound, high-frequency noise
	Overheating	Motor overheating, prolonged use	Increased noise, excessive vibration
	Blade damage	Broken blades, physical damage	Low-frequency vibration, unsteady sound
Pump	Rotational imbalance	Imbalanced shaft, misalignment	Vibration, high-frequency noise
	Mechanical wear	Component wear, friction	Increased noise, irregular sound
	Leakage	Seal damage, loose connections	Abnormal fluid sound, flow irregularities
	Bearing damage	Bearing wear, friction	High-frequency noise, unstable sound pattern

**Table 4:** VAE-based fault diagnosis results obtained using the MIMII dataset corresponding to nonrotating equipment.

Machine	SNR	Case A: MFCC (Mean, Max, Min)	Case B: MSS Enhancement	Case C: Signal Trans. Augmentation	Case D: GAN Augmentation
Non-rotary equipment	6 dB	0.9227	0.9927	0.9894	0.9885
	0 dB	0.8348	0.9499	0.9554	0.9510
	−6 dB	0.7164	0.8586	0.8876	0.8517

**Table 5:** VAE-based fault diagnosis results obtained using the MIMII dataset corresponding to rotating equipment.

Machine	SNR	Case A: MFCC (Mean, Max, Min)	Case B: MFCC Lightweighting	Case C: Signal Trans. Augmentation	Case D: GAN Augmentation
Rotary equipment	6 dB	0.9769	0.9782	0.9870	0.9774
	0 dB	0.9125	0.9175	0.9284	0.9083
	−6 dB	0.7742	0.7816	0.8052	0.7650

**Table 6:** VAE-based fault diagnosis results obtained using forklift equipment.

Machine	SNR	Case A: MFCC (Mean, Max, Min)	Case B: MSS Enhancement	Case C: Signal Trans. Augmentation	Case D: GAN Augmentation
Forklift	6 dB	0.9544	0.9667	0.9551	0.9465
	0 dB	0.8827	0.9366	0.9155	0.8973
	−6 dB	0.8159	0.8453	0.8469	0.8085

**Table 7:** Average F1-scores corresponding to the DANN-based fault diagnosis results of MIMII nonrotating equipment.

Machine	Case 1		Case 2		Case 3	
	Before	After	Before	After	Before	After
Non-rotary equipment	77.37%	97.03%	64.84%	87.57%	72.46%	91.45%

**Table 8:** DANN-based fault diagnosis results obtained from MIMII rotating equipment.

Machine	Case 1:		Case 2:		Case 3:	
	Before	After	Before	After	Before	After
Rotary equipment	85.12%	96.52%	62.18%	84.47%	78.72%	91.38%

**Table 9:** DANN-based fault diagnosis results obtained from a forklift.

Machine	Case 1:		Case 2:		Case 3:	
	Before	After	Before	After	Before	After
Forklift	95.14%	95.99%	78.82%	80.89%	93.04%	96.12%

## Data Availability

The MIMII dataset used in this study can be accessed through the DOI provided in reference [1]: https://arxiv.org/abs/1909.09347. The forklift datasets presented in this article are not readily available because they were measured using equipment that is part of corporate assets and therefore cannot be publicly disclosed. Requests to access the datasets should be directed to the corresponding author (jh_lee@inha.ac.kr).
